# Evaluating Prey Availability for the Rice's Whale (*Balaenoptera ricei*) Based on Environmental DNA

**DOI:** 10.1002/ece3.72789

**Published:** 2026-01-13

**Authors:** Katherine Silliman, Lynsey A. Wilcox Talbot, Mary Applegate, Laura Aichinger Dias, Lance P. Garrison, Mark Grace, Corinne N. Paterson, Luke R. Thompson, Nicole L. Vollmer, Patricia E. Rosel

**Affiliations:** ^1^ Ocean Chemistry and Ecosystems Division, Atlantic Oceanographic and Meteorological Laboratory, National Oceanic and Atmospheric Administration Miami Florida USA; ^2^ Northern Gulf Institute Mississippi State University Starkville Mississippi USA; ^3^ Marine Mammal and Turtle Division, Southeast Fisheries Science Center, National Marine Fisheries Service, National Oceanic and Atmospheric Administration Lafayette Louisiana USA; ^4^ Cooperative Institute for Marine and Atmospheric Studies, Rosenstiel School for Marine, Atmospheric, and Earth Science University of Miami Miami Florida USA; ^5^ Marine Mammal and Turtle Division, Southeast Fisheries Science Center, National Marine Fisheries Service, National Oceanic and Atmospheric Administration Miami Florida USA; ^6^ Tulane University Department of Ecology and Evolutionary Biology New Orleans Louisiana USA

**Keywords:** eDNA metabarcoding, fish biodiversity, prey availability, Rice's whale

## Abstract

Understanding the foraging ecology of endangered marine mammals provides important information for their conservation yet remains challenging due to the elusive, underwater nature of their feeding habits. Here, we used environmental DNA (eDNA) metabarcoding with two complementary 12S rRNA markers to characterize potential prey communities available to the critically endangered Rice's whale (*Balaenoptera ricei*) in its core habitat in the northeastern Gulf of America (formerly Gulf of Mexico). Water samples (*N* = 21) collected during a 2019 survey within Rice's whale feeding areas detected 99 unique fish species across 62 families, exceeding the diversity recorded by concurrent trawl surveys. The combined metabarcoding approach revealed 74 fish species not recorded in trawls, while 16 trawl‐caught species went undetected by eDNA. Notably, eDNA yielded higher detection rates for several potential prey taxa previously identified through stable isotope analysis and trawl surveys, resulting in an updated list of top potential prey. These findings suggest that key prey species may be more prevalent in Rice's whale habitat than previously documented. To support these analyses, existing reference databases were expanded by sequencing the 12S rRNA gene from 15 regional fish species, using new primers developed for this study. Our study demonstrates the value of eDNA as a complementary tool for monitoring the prey community of this critically endangered cetacean, while highlighting the need for continued development of reference databases to maximize the ecological insights gained from marine metabarcoding applications.

## Graphical abstract

To characterize the prey community of the critically endangered Rice's whale, we employed environmental DNA (eDNA) metabarcoding in its core habitat. Our multi‐marker approach detected significantly greater fish biodiversity than concurrent trawl surveys and revealed a higher prevalence of key prey taxa. These results indicate that the foraging base for Rice's whales may be more diverse and widespread than previously documented, providing valuable data for the species' conservation.

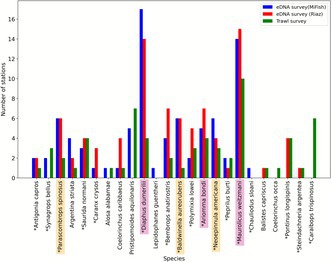

## 1 Introduction

Understanding predator–prey interactions in marine ecosystems provides valuable insights for conservation and management, particularly for endangered species with restricted ranges. However, studying the foraging ecology of marine mammals remains challenging, as their mobility, deep diving behavior, and the remote nature of their feeding habitats make direct observation difficult (Nelms et al. 2021). The Rice's whale (*Balaenoptera ricei*), with an estimated population size of only ~51 (CV = 0.50) individuals (Garrison 2020; Hayes et al. 2023) and listed as critically endangered on the IUCN Red List (Rosel et al. 2022), is endemic to the Gulf of America (formerly Gulf of Mexico) (Rosel et al. 2021; Rosel and Garrison 2022). Only recently recognized as a separate species (Rosel et al. 2021), very little is known about the biology and ecology of Rice's whales. In the heavily industrialized waters of the Gulf, the species faces multiple anthropogenic threats, including vessel strikes, underwater noise from vessels and energy exploration activities, fisheries interactions, and ingestion of marine debris (Rosel et al. 2016; Soldevilla et al. 2017; Aichinger Dias et al. 2025).

Similar to other rorqual whales, Rice's whales forage by lunge‐feeding and are thought to feed on pelagic schooling fish (Kok et al. 2023; Shadwick et al. 2019). Rice's whales, however, exhibit a unique feeding strategy in which they perform foraging dives primarily during the day at depths near the seafloor, whereas during the night, the whales spend more time at the sea surface, performing fewer or no foraging lunges (Kok et al. 2023). This behavior indicates that they prey on diel vertically migrating organisms. A recent study leveraged stable isotope analysis and fish trawl surveys to provide the first insight into the whale's diet and prey availability. Kiszka et al. (2023) found that Rice's whales may be actively selecting schooling prey with high energy content, but overall our understanding of their prey selection and feeding ecology remains limited due to the lack of stomach content samples or direct feeding observations. Furthermore, as climate and environmental changes have the potential to affect prey species, and impacts associated with energy exploitation in the region continue, understanding and managing these threats may improve the recovery of this small and isolated population.

Environmental DNA (eDNA) metabarcoding has emerged as a powerful tool for monitoring marine biodiversity and understanding species distributions (Djurhuus et al. 2020; Valentini et al. 2016). This approach detects DNA shed by organisms into the water column through scales, mucus, excrement, and other cellular material, providing a non‐invasive method to assess species presence and relative abundance. When combined with traditional sampling methods, eDNA can offer complementary insights into marine community composition and predator–prey relationships (Boyse et al. 2024; Qu et al. 2020; M. Riaz et al. 2023). Recent studies have demonstrated concordance between eDNA detections and traditional fish surveys, in many cases showing that eDNA can be more sensitive and comprehensive than trawl surveys (Cote et al. 2023; Gold et al. 2022; Thomsen et al. 2016).

The choice of genetic markers for metabarcoding significantly influences which taxa can be detected and reliably identified (García‐Machado et al. 2023; Kumar et al. 2022). For marine vertebrates, mitochondrial gene markers are commonly used due to their high copy number, taxonomic resolution, and available reference sequences. However, these markers vary in their amplification efficiency and ability to resolve species‐level identifications (Fontes et al. 2024; Marques et al. 2021; Turner et al. 2014). The 12S rRNA region, in particular, has shown strong performance for fish species detection, with multiple mitochondrial markers having been developed from this gene (Kelly et al. 2014; Miya et al. 2015; Riaz et al. 2011). Using multiple markers can help overcome the taxonomic biases of a single marker and provide a more comprehensive view of marine community composition. Even so, the utility of metabarcoding data still depends on the completeness of reference sequence databases. These databases remain patchy for many marine taxa, particularly in understudied regions and for non‐commercial species (Gold et al. 2021; Marques et al. 2021; Stoeckle et al. 2020). Expanding these reference libraries through targeted sequencing efforts is essential to improve taxonomic assignment accuracy and maximize the ecological insights from eDNA surveys.

In this study, we leveraged eDNA metabarcoding of two 12S markers (Riaz and MiFish; see methods) and expanded the reference library with sequences from local fish species to investigate the potential fish prey available to the Rice's whale in its core habitat in the northeastern Gulf of America. By analyzing water samples collected during a field survey targeting Rice's whale feeding areas, we aimed to compare fish community composition detected through eDNA versus traditional trawl surveys in order to evaluate the utility of eDNA as a complementary tool for characterizing Rice's whale prey species. Our findings provide new insights into the prey community available to the Rice's whale while demonstrating the value of integrating multiple sampling approaches to understand the foraging ecology of endangered marine mammals.

## 2 Methods

### 2.1 Sample Collection

During July 2019, fish trawls (31.7 m footrope length, 0.6 cm sq. codend mesh liner) were deployed from the NOAA Ship *Gordon Gunter* to collect potential Rice's whale fish prey during daylight hours within known feeding areas in the northeastern Gulf of America (Kiszka et al. 2023; Martinez et al. 2025). Trawl stations were selected based on near‐bottom backscatter signals from aggregations of organisms (i.e., fishes) detected using a Simrad EK80 echosounder (transducer frequencies 18 kHz, 38 kHz, and 120 kHz). When potential Rice's whale prey aggregations were located, the trawl was deployed typically to a trawl depth 5 m above the sea bottom. Specimens captured from trawls were identified and sorted by species, measured, weighed, and cataloged. Representative voucher specimens from each taxon were tissue sampled for various analyses, with tissues stored in 95% ethanol at −20°C. Trawling efforts captured 35,606 specimens represented by the taxa: Anguilla, Annelida, Arthropod, Cephalopod, Cnidaria, Echinoderm, Elasmobranch, Mollusca, Polychaete, Teleost, and Tunicate. Three hundred and eighteen specimens were sampled for analyses that variously included DNA barcoding, stable isotopes, contaminants, or calorimetry. Additional details of trawling procedures and information on the sampling stations can be found in Kiszka et al. (2023) and Martinez et al. (2025).

**FIGURE 1:**
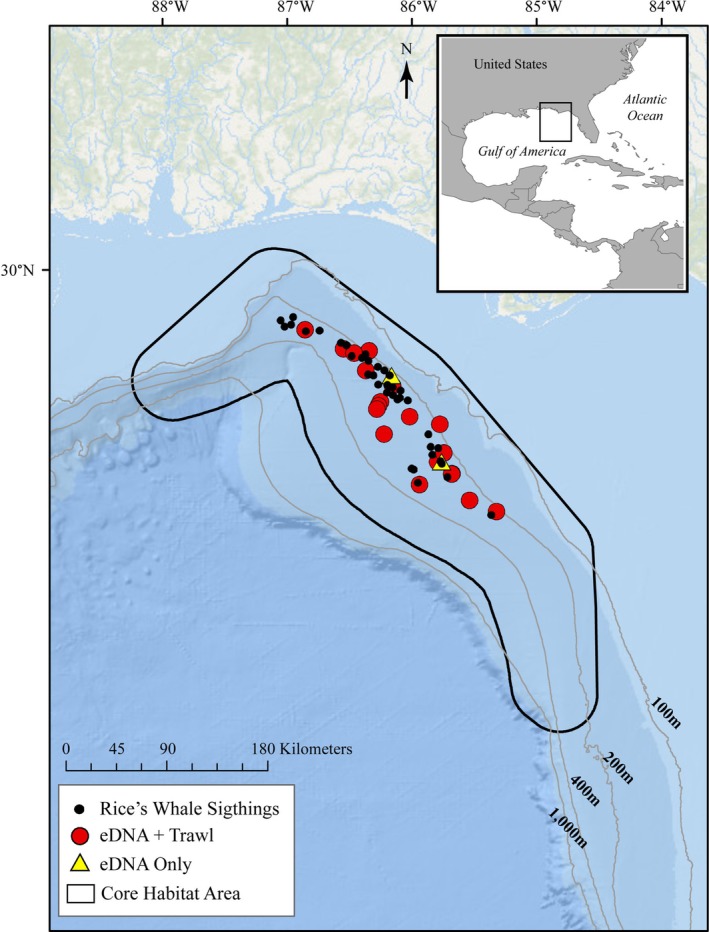
Map of eDNA sampling stations and Rice's whale sightings within the species' core habitat (black outline) in the Gulf of America during July 2019 surveys. Symbols indicate eDNA collections with concurrent trawling (red dots), eDNA collection during whale sightings only (yellow triangles), and visual sightings of Rice's whales (black dots).

A total of 21 seawater samples were collected between 120 and 320 m depth concurrently with the trawling, using Niskin bottles (5 L total capacity) attached to a conductivity, temperature, and depth sensor (CTD) unit (Figure 1). Bottles were triggered to close via remote command at the desired sampling depth (Table [Link], [Link]). CTDs were deployed for seawater collection immediately after the trawl was retrieved at 18 stations. At 17 stations, a sample was collected from a single target depth. At one station, samples were collected from two different depths (Table S1). Two additional CTD deployments collected seawater when no trawling was occurring while Rice's whales were in the immediate vicinity and displaying feeding behavior (Table S1).

All Niskin bottles were sterilized before use by thoroughly rinsing several times with ~500 mL of 20% bleach for 10 min, followed by multiple rinses with tap water, and allowed to air dry. The outside of each bottle was wiped with a Clorox Disinfecting Wipe and allowed to air dry. After each sample was collected, approximately 2 L of seawater was transferred from the Niskin bottle into a 2 L sterile Nalgene bottle (cleaned as described for the Niskin bottles prior to use) and placed into a cooler with ice until filtration. All samples were filtered within 35 min of collection. Filtration, cleaning, and sample storage followed that as described in Wilcox Talbot et al. (2025), using a 47 mm diameter mixed cellulose ester (MCE) filter with a 0.45‐μm pore size. Using sterile forceps, each MCE filter was placed in a 4 mL screw cap vial containing approximately 3 mL of sterile Longmire's lysis buffer (Longmire et al. 1997). The preserved filters were stored at room temperature and in the dark until eDNA extraction. Longmire's buffer was chosen as a preservative at the time of sampling to allow filter storage at room temperature in the field, for continuity with previously collected eDNA samples from 2018 (Wilcox Talbot et al. 2025), and based on a prior successful eDNA study (Parsons et al. 2018). For negative field controls, Nalgene bottles were used to collect 2–2.8 L of ship's water, and filtered using the same process as completed for a Niskin seawater sample. To avoid cross‐contamination, all eDNA sampling preparation and processing were conducted isolated from trawling activities and specimens. Staff who processed the eDNA samples did not assist in sorting trawl specimens and vice‐versa. In addition, eDNA processing was performed in locations on the ship where no tissue samples were handled.

### 2.2 Molecular Methods

#### 2.2.1 Voucher Tissue DNA Extractions and Sequencing

Genomic DNA was extracted from muscle tissue or fin clips of 15 voucher species collected from the trawls using a DNeasy Blood and Tissue kit (Qiagen), quantified with a Qubit 4 Fluorometer (Thermo Fisher Scientific), and quality‐checked via gel electrophoresis. The mitochondrial 12S rRNA gene was sequenced using new primers designed for this study: forward primer PHE_FishF (5′‐CATAACACTGAAGATGTTAAG‐3′) with reverse primer VAL_FishR (5′‐TYTCAGTGTAAGGGARATGC‐3′) for all species except *Lepidopus altifrons* , in which the forward primer PHE_FishF2 (5′‐TCACTGAAGATGWYAAG‐3′) was used with VAL_FishR (Table S2). These primers were designed to target the flanking phenylalanine and valine tRNA regions. Conserved regions (> 20 bp) were identified by aligning sequences from GenBank for the voucher species or their close relatives (Table S3) using Geneious Prime 2021 (https://www.geneious.com). These regions were further validated against a taxonomically broad alignment generated via nucleotide BLAST (Altschul et al. 1990) of the *Ariomma indica* mitochondrial genome (GenBank AP012513), selected because *A. bondi* is a key prey species for Rice's whales (Kiszka et al. 2023). The 3′ end of each primer was anchored with at least one G/C nucleotide to strengthen annealing. As *L. altifrons* failed to amplify with the standard set, a specific forward primer (PHE_FishF2) was designed using *Assurger anzac* as a phylogenetic proxy. Annealing temperatures were optimized via a gradient PCR (45°C, 48°C, 52°C, 55°C) on a subset of fish species.

Final PCR reactions (25 μL) contained 20 mM Tris–HCl (pH 8.4), 50 mM KCL, 1.5 mM MgCl_2_, 150 μM dNTPs, 1 U *Taq* DNA Polymerase (Invitrogen), 0.3 μM of each primer, and 4 to 25 ng of DNA. Cycling conditions for the PHE_FishF and VAL_FishR primer set were: 95°C for 2 min; 35 cycles of 95°C for 45 s, 55°C for 45 s, and 72°C for 45 s; with a final extension at 72°C for 7 min. Cycling conditions for the PHE_FishF2 and VAL_FishR amplification were the same except for an annealing temperature of 48°C. A negative PCR control was included in each amplification set. Amplification products were verified by agarose gel electrophoresis and purified via low melting point agarose extraction with agarase (Sigma‐Aldrich). The PHE_FishF/VAL_FishR products were sequenced using a BigDye Terminator v1.1 Cycle Sequencing Kit (Thermo Fisher Scientific) and run on an ABI 3500 Genetic Analyzer. Due to equipment unavailability at the time, the PHE_FishF2/VAL_FishR products were sequenced commercially (Eurofins Genomics) using BigDye Terminator v3.1 on an ABI 3730xl Genetic Analyzer. Forward and reverse sequences were aligned and assembled into consensus sequences using Geneious Prime 2024.0.4. The resulting reference sequences were added to the NCBI GenBank database (https://www.ncbi.nlm.nih.gov/genbank; Table S2).

#### 2.2.2 eDNA Extractions

eDNA was extracted from 21 CTD seawater samples and 3 negative field controls within 4 months of water collection and filtering following methods described in Wilcox Talbot et al. 2025 and summarized here. The extractions were performed in a trace DNA laboratory, in which no tissue samples or post‐PCR products are handled. All surfaces and equipment were sterilized with 10% bleach and/or DNA AWAY surface decontaminant, and pipettes, trays, and tubes were irradiated with UV light for at least 15 min prior to extractions. The 4 mL screw cap vials containing each filter and Longmire's lysis buffer were incubated at 65°C for 30 min on a shaking platform with periodic mixing by vortexing for 30 s. All of the Longmire's lysis buffer (~3 mL) from each vial was then transferred to a 15 mL conical tube. An equal volume of phenol:chloroform (1:1) was added to each and mixed by inversion for 1 min, followed by centrifugation for 10 min at 5000 rpm. The aqueous layer above the interface was transferred to a new tube and a second wash was performed with an equal volume of chloroform only. The aqueous layer was transferred to a new conical tube and DNA was precipitated with 1/24 volume of 5 M NaCl and 2× the volume of ice‐cold 100% ethanol. After mixing by inversion, the tubes were incubated at −20°C overnight. Then, all of the liquid was transferred to 2‐mL low adhesion tubes and the DNA was pelleted by centrifugation for 10 min at 15,000 rpm. Pellets were washed with 500 μL of ice‐cold 100% ethanol followed by 500 μL of ambient temperature 70% ethanol before drying in an incubator at 45°C for 15–30 min. The eDNA was resuspended using 1× TE Buffer, low EDTA (10 mM Tris, 0.1 mM EDTA) for a final volume of 200 μL and stored at −80°C. A control of Longmire's lysis buffer was processed with each extraction set for a total of four extraction controls. The quality of the extracted eDNA was examined via agarose gel electrophoresis and quantity was measured using a Qubit 4 Fluorometer. The eDNA extractions were cleaned using a OneStep PCR Inhibitor Removal kit (Zymo Research) prior to PCR amplification following the manufacturer's protocol.

### 2.3 eDNA Metabarcoding

The 12S rRNA gene was amplified using two different primer sets for eDNA metabarcoding of fish species: modified MiFish‐U‐F/R2 primers (Miya et al. 2015; Stoeckle et al. 2022) and the Riaz primers (Riaz et al. 2011). Primers were constructed with Fluidigm common oligos CS1 forward and CS2 reverse fused to their 5′ ends, to enable two‐step library preparation at the Michigan State University Research Technology Support Facility (RTSF) (Table 1). Each PCR reaction had a total volume of 25 μL and contained 0.25 μL of Phusion Plus DNA Polymerase (Thermo Fisher Scientific), 1× Phusion Plus Buffer, 0.2 μM each primer with Fluidigm adaptor, 0.2 mM dNTPs, 3 μL of eDNA, and 0.08 mg/mL bovine serum albumin (BSA, Sigma‐Aldrich). Amplification was performed using an initial denaturation step of 98°C for 30 s followed by 40 cycles of 98°C for 20 s, 60°C for 20 s, and 72°C for 20 s with a final extension step at 72°C for 5 min. Negative PCR controls were included in each amplification set by adding sterile ultrapure water in place of eDNA, for a total of six PCR controls. Five μL of each reaction were run on a 2% agarose gel to assess amplification. Six PCR replicates were performed for each eDNA sample and field control and the PCR products were pooled for each sample separately. All PCR negative controls were pooled together. The four extraction controls were each amplified once and the products were not combined. All pools were cleaned using a MinElute PCR Purification Kit (Qiagen), eluted with 25 μL of Buffer EB (10 mM Tris·Cl, pH 8.5), quantified via Qubit 4 Fluorometer, and verified on 2% agarose gel. The cleaned PCR products were diluted to 10 ng/μL in sterile ultrapure water and 100 ng of each was sent to the Michigan State University Research Technology Support Facility Genomics Core for library preparation and sequencing. The Genomics Core performed secondary PCR using dual‐indexed, Illumina‐compatible primers targeting the Fluidigm CS1/CS2 oligomers at the ends of the primary PCR products (Table 1). Amplicons were batch normalized using Invitrogen SequalPrep DNA Normalization plates and recovered product was pooled separately for the Riaz and MiFish samples. Blue Pippin Prep size selection was performed on the MiFish pool to isolate the desired 350 base pair (bp) product from a 190 bp non‐target product. The pools were quality controlled (QC) and quantified using a combination of Qubit dsDNA HS, Agilent 4200 TapeStation HS DNA1000 and Invitrogen Collibri Library Quantification quantitative PCR (qPCR) assays. The two library pools were then combined in equimolar amounts for sequencing, followed by final qPCR quantification. Sequencing was carried out on an Illumina MiSeq in a 2 × 250 bp paired end format using a MiSeq v2 500 cycle reagent cartridge. Base calling was done by Illumina Real Time Analysis (RTA) v1.18.54 and output of RTA was demultiplexed and converted to FastQ format with bcl2fastq v2.20.0, Illumina Inc.

**TABLE 1:** Primer sets, with Fluidigm adapters (underlined), and details on marker‐specific custom reference databases used.

Primer name	Primer ID	Primer sequence (5′‐3′)	Amplicon size (avg.)	Species in database	Unique sequences in database	Citation
MiFish	MiFish_U Forward MiFish_U Reverse2	ACACTGACGACATGGTTCTACAGTCGGTAAAACTCGTGCCAGC TACGGTAGCAGAGACTTGGTCTCATAGTGGGGTATCTAATCCCAGTTTGT	170 bp	9181	16,969	Miya et al. (2015); Stoeckle et al. (2022)
Riaz	Riaz_Forward Riaz_Reverse	ACACTGACGACATGGTTCTACAACTGGGATTAGATACCCC TACGGTAGCAGAGACTTGGTCTTAGAACAGGCTCCTCTAG	96 bp	6823	11,738	T. Riaz et al. (2011)

### 2.4 Bioinformatics and Data Analysis

Raw sequencing reads were first trimmed of forward and reverse primer sequences using cutadapt v4.4, discarding reads that were missing the forward primer and less than 50 bp in length (Martin 2011). Trimmed reads were then processed separately for each marker with Tourmaline v1.1.1, which implements QIIME2 (v2023.5) and DADA2 plugins in a Snakemake workflow (Thompson et al. 2022; Bolyen et al. 2019). Paired‐end DADA2 was used to infer MiFish and Riaz amplicon sequence variants (ASVs) (Callahan et al. 2016). Singleton ASVs were removed. Taxonomic assignments were also performed in Tourmaline using Naïve Bayes classifiers trained on custom reference sequence databases for each marker (Bokulich et al. 2018). The MiFish reference sequence database included a previously published rCRUX‐generated database leveraging NCBI nucleotide database (nt) sequences as of December 2022 (Gold, Curd, et al. 2023), sequences from the MitoFish database (Zhu et al. 2023) missing from the rCRUX database, 148 recently generated sequences across 68 Gulf fish taxa (Janosik et al., in review), and the 15 reference fish sequences generated in this study (available at https://doi.org/10.5281/zenodo.17664846). The Riaz reference sequence database included a newly developed rCRUX database leveraging NCBI nt sequences as of September 2024 (Curd et al. 2023), sequences from MitoFish, 160 recently generated sequences across 70 Gulf fish taxa (Janosik et al., in review), and the 15 newly generated reference sequences in this study (available at https://doi.org/10.5281/zenodo.17727349). Both databases were filtered to only include bony fish, elasmobranchs, and mammals. The MiFish database had 16,969 sequences across 9181 unique species and 3049 unique genera, while the Riaz database had 11,738 sequences across 6823 unique species and 2841 unique genera (Table 1). Taxonomy assignments were verified by BLAST against NCBI nt (October 2024) and curated for regional accuracy. Species absent from the Gulf of America were reassigned to the genus level. Furthermore, sequences matching multiple species with 100% similarity were disambiguated by assigning them to the species native to the Gulf region. Downstream filtering of taxa data, ASV tables, and read counts were performed in Python. Detections of taxa less than 1/1000 of the total for that amplicon sequence variant (ASV) across all samples in the MiSeq run were excluded to reduce mis‐assignment (Stoeckle et al. 2020). Read counts were summed by taxonomic assignment and normalized by the total library size of each sample to calculate relative read abundance (Deagle et al. 2019). For biodiversity comparisons with trawl data, only Actinopteri and Chondrichthyes taxa were retained, and taxa unannotated at the order level or higher were removed.

Concurrent trawl data collected during the survey were filtered to remove non‐fish taxa and only include stations where eDNA samples were also taken for comparison of presence‐absence and taxonomic richness between methods. Stable isotope data for 35 fish species based on samples taken during the concurrent trawls were obtained from Kiszka et al. (2023). That study identified three fish species ( *Ariomma bondi* , *Diaphus dumerilii* , and *Maurolicus weitzmani* ) and one squid genus (*Doryteuthis* sp.) as the most likely Rice's whale prey items, with δ13C values ranging from −19.53 to −16.14 and δ15N values from 9.36 to 12.68. In our current study, top potential Rice's prey were determined as all fish species with isotope values within this range, as well as eDNA detected in at least 5 stations (> 25%) and an average metabarcoding relative read abundance of > 6% for at least one marker across stations where they were detected. Habitat and depth range were obtained from Fishbase for these top potential prey species.

## 3 Results

### 3.1 Sequencing and Bioinformatics

The 12S rRNA gene was successfully sequenced from 36 voucher fish specimens, adding 15 species from the Gulf of America to the custom reference databases (GenBank accession number PV861998‐PV862034; Table S2). After trimming of low quality base pairs and annotating the gene region, 3 *L. altifrons* specimens had complete 12S rRNA sequences and the rest of the species had partial coverage of at least 95%.

Metabarcoding of the MiFish marker resulted in an average of 205,092 raw reads per sample and 252 raw reads per control sample. After removing primers, filtering and trimming for quality, merging paired reads, and removing chimeras, 77% of the raw sample reads were retained across 245 ASVs for downstream analyses (average 161,035 MiFish reads per sample). For the Riaz marker, an average of 258,598 raw reads per sample was obtained, of which 89% across 134 ASVs were used for downstream analyses. For the Riaz control samples, field blanks averaged 131,930 reads dominated by *Homo sapiens* (average 86% of reads), compared to an average of only 183 reads for extraction blanks and PCR negative controls. Taxonomic assignment rates were similar across markers, with 74% of MiFish ASVs and 78% of Riaz ASVs assigned to species‐level resolution.

### 3.2 Biodiversity of eDNA and Trawling Methods

**FIGURE 2:**
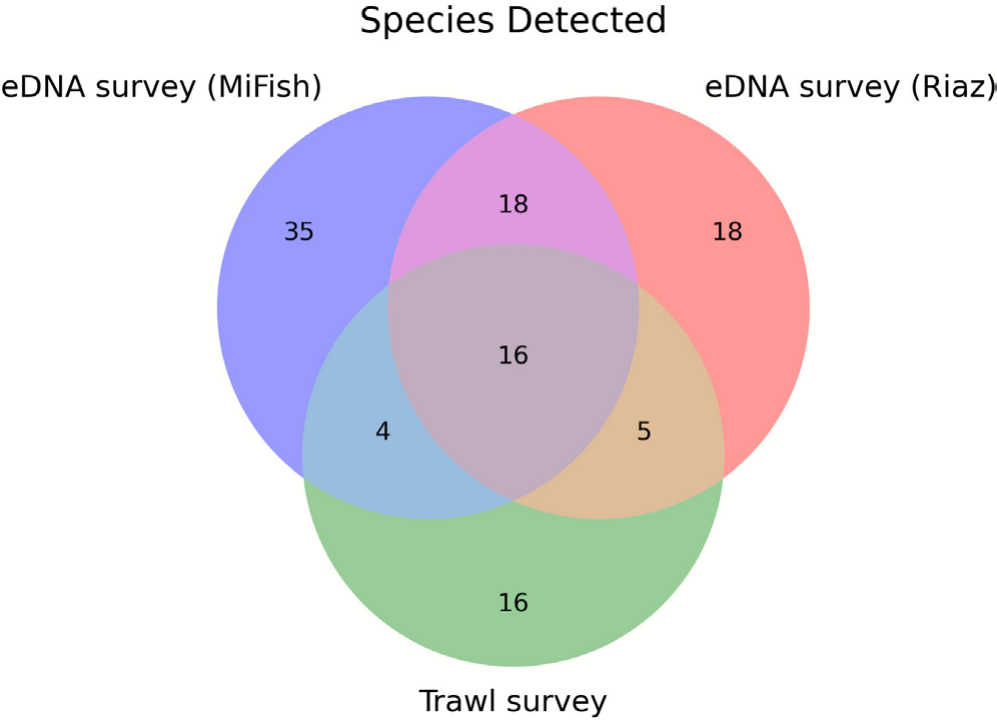
Overlap of fish species detections between trawl and eDNA surveys, limited to stations where both data types were collected.

**FIGURE 3:**
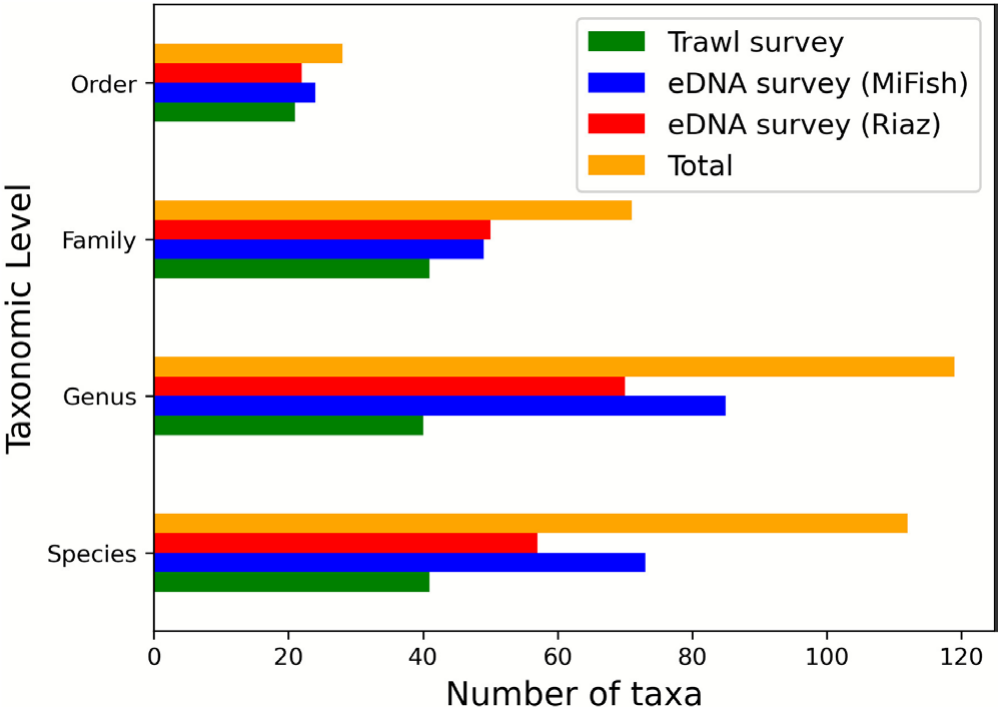
Comparison of taxonomic richness of fish species detections between trawl and eDNA surveys, limited to stations where both data types were collected.

**FIGURE 4:**
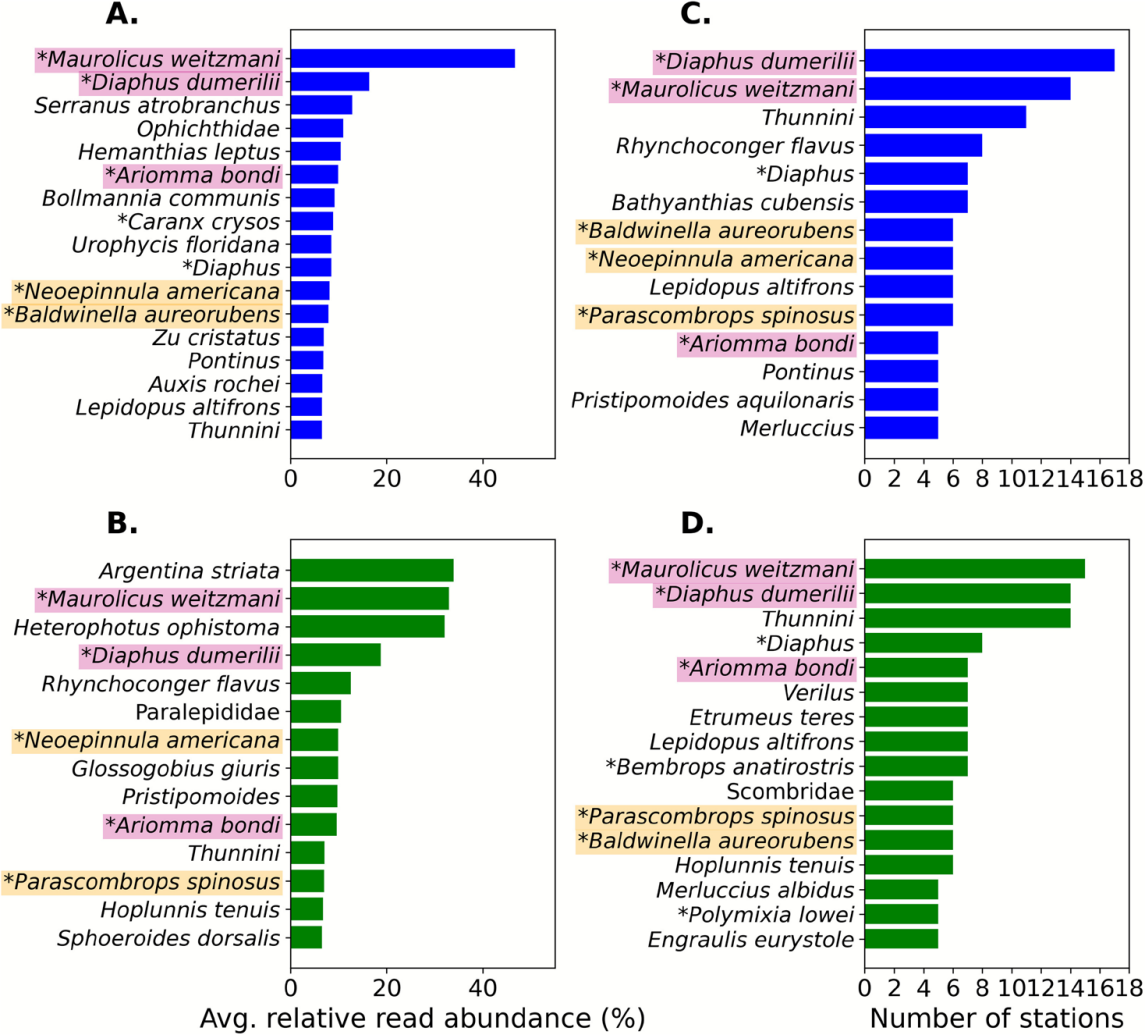
Bar charts displaying the taxonomic units with average relative read abundances > 6% (excluding non‐detections) for (A) MiFish marker and (B) Riaz marker. Taxonomic units prevalent in > 25% of stations are shown for (C) MiFish marker and (D) Riaz marker. Taxa with an (*) have isotope values within the range of previously described Rice's whale prey taxa. Taxa highlighted in pink were originally identified as top potential prey in Kiszka et al. (2023), while taxa in yellow are new suggestions as potential prey from this study.

**FIGURE 5:**
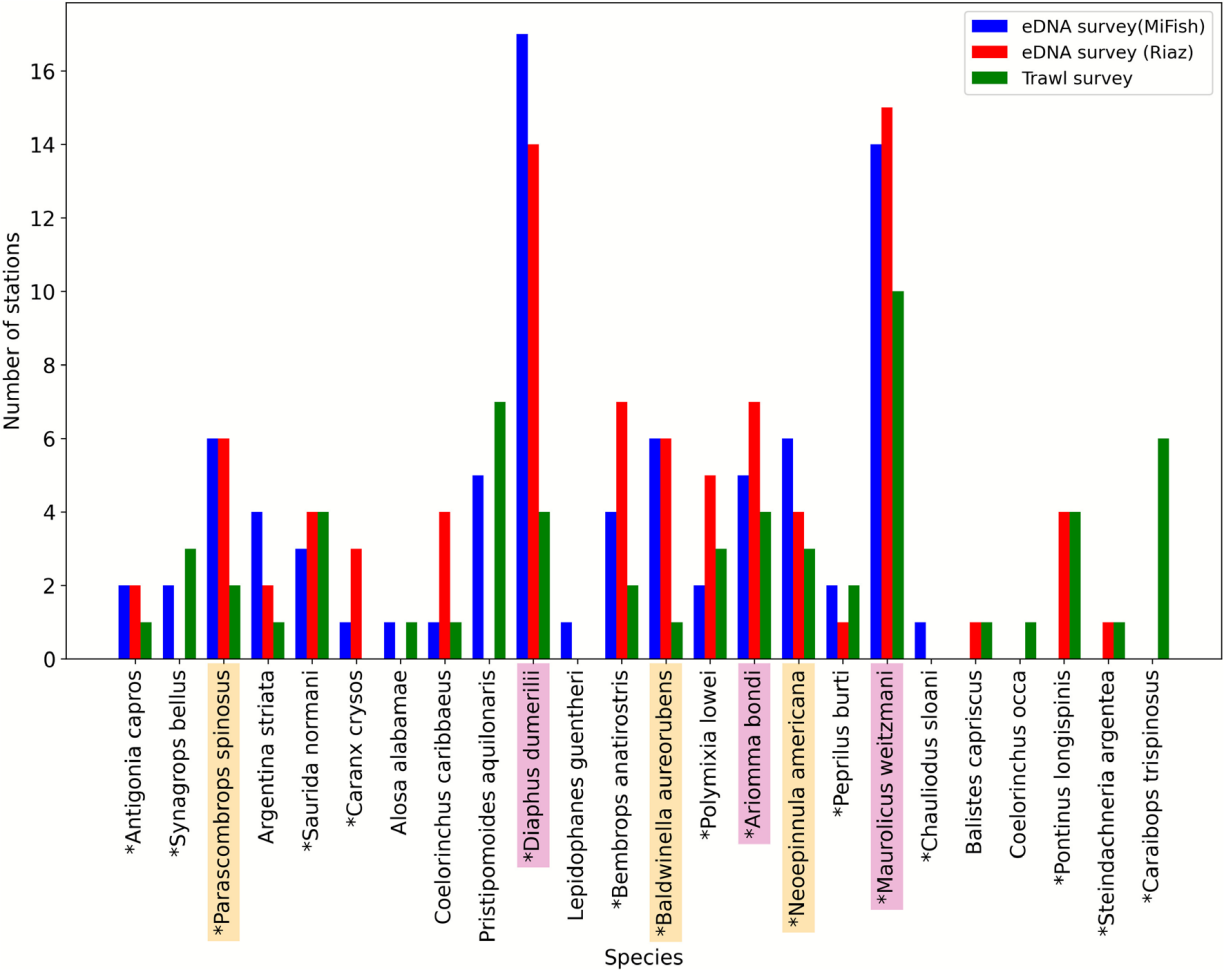
Occurrence of fish taxa with available stable isotope data for trawl and eDNA surveys with the MiFish eDNA marker and the Riaz eDNA marker. Taxa with an (*) have stable isotope values within the range of previously described Rice's whale prey taxa. Taxa highlighted in pink were originally identified as top potential prey in Kiszka et al. (2023), while taxa in yellow are new suggestions of potential Rice's whale prey based on eDNA sampling from this study. Only stations where both eDNA and trawl data were collected are reported.

eDNA methods consistently detected higher taxonomic richness than the trawl sampling (Figures 2 and 3). While trawling captured 41 fish species, the combined use of the MiFish and Riaz markers at the same paired stations identified a total of 96 unique fish species, 106 genera, and 61 families. Of the new local species added to our custom reference database to improve these assignments, all but one were detected by at least one eDNA marker; the single undetected species (angelshark, *Squatina dumeril* ) is not a known prey item for rorqual whales. Comparisons between the two markers revealed distinct differences in detection capabilities. The MiFish marker detected a higher number of species (73) compared to the Riaz marker (57) (Figure 2). While there was overlap, with 34 species and 38 families detected by both markers, the sets were largely complementary: 62 species and 23 families were detected by only one of the two markers. These results were qualitatively similar when looking across all 21 eDNA samples, including those taken when Rice's whales were observed feeding (Figures S1 and S2). In terms of community composition, the three most prevalent taxa across stations for both markers were *M. weitzmani* , *D. dumerilii* , and sequences resolved to the tribe Thunnini (tunas). Based on average relative read abundance (excluding non‐detections), *M. weitzmani* was the dominant taxon for both markers (Figures 4 and 5).

Of the 41 fish species recorded from the trawl survey at these stations, 16 were not detected by either marker (Figure 1). Conversely, eDNA metabarcoding identified 74 marine fish species that were not observed in the trawls, including species of commercial importance such as the blue runner ( *Caranx crysos* ), Northern red snapper ( *Lutjanus campechanus* ), and king mackerel ( *Scomberomorus cavalla* ). Although both the Riaz and MiFish primers have been recorded to occasionally identify marine mammals, we did not observe Rice's whale eDNA in our data despite the reference sequence being present in the reference database. However, we did observe common bottlenose dolphin ( *Tursiops truncatus* ) and rough‐toothed dolphin ( *Steno bredanensis* ) eDNA using the Riaz marker.

### 3.3 Characterizing Potential Prey Species

Potential fish prey species were identified based on eDNA‐derived relative read abundance and prevalence in the foraging habitat, as well as stable isotope values within −19.53 to −16.14 for δ13C and 9.36 to 12.68 for δ15N, as determined in Kiszka et al. (2023). The top three species based on these criteria were the same as identified in Kiszka et al. (2023): *M. weitzmani, D. dumerilii*, and *A. bondi* . Three additional species emerged as potential prey: *Parascombrops spinosus*, *Baldwinella aureorubens*, and *Neoepinnula americana* (Table 2, Figures 4, 5). Among the 17 fish species with isotope values matching the range of potential Rice's whale prey, 11 were detected more frequently by at least one eDNA marker compared to trawl surveys (Figure 5).

**TABLE 2:** Potential fish prey species of Rice's whale inferred from eDNA data, including percent occurrence in eDNA samples (*N* = 21) and in trawl samples (*N* = 18), average percent relative read abundance (excluding non‐detections), mean (±SD) stable carbon (δ13C) and nitrogen (δ15N) isotope values (from Kiszka et al. 2023), habitat, and depth range. Habitat and depth range taken from Fishbase.

Species	Common name	eDNA occurrence (MiFish/Riaz)	Trawl occurrence	eDNA read abundance (MiFish/Riaz)	δ13C ± SD, δ15N ± SD	Habitat and depth range
*Maurolicus weitzmani*	Atlantic pearlside	81.0%/86.0%	55.6%	52.4%/39.1%	−19.22 ± 0.09, 10.64 ± 1.02	Pelagic, Bathypelagic 0–100 m
*Diaphus dumerilii*	Dumeril's lanternfish	95.2%/71.4%	22.2%	14.2%/17.5%	−18.83 ± 0.24, 10.63 ± 1.27	Pelagic‐oceanic 0–805 m
*Ariomma bondi*	Silver‐rag driftfish	23.8%/38.1%	22.2%	9.9%/8.8%	−18.34 ± 1.24, 10.66 ± 1.24	Demersal 50–500 m
^*a*^ *Neoepinnula americana*	American sackfish	33.3%/19.1%	16.7%	6.9%/9.9%	−18.08 ± 0, 12.75 ± 0.82	Bentho‐pelagic 184–457 m
^*a*^ *Baldwinella aureorubens*	Streamer bass	28.6%/28.6%	5.6%	7.8%/0.5%	−18.4 ± 0, 12.36 ± 0.11	Demersal 120–610 m
^*a*^ *Parascombrops spinosus*	Keelcheek bass	33.3%/33.3%	11.1%	4.7%/6.1%	−17.67 ± 0.02, 12.65 ± 0.06	Bathydemersal 87–544 m

^a^ New potential prey species identified by this study are marked.

## 4 Discussion

Understanding prey availability and distribution provides essential information for the conservation of endangered marine mammals, particularly for species with restricted ranges and specialized feeding behaviors like the Rice's whale. Our study demonstrates that eDNA metabarcoding provides a more comprehensive assessment of potential prey communities available to this critically endangered cetacean when combined with traditional trawl surveys. Furthermore, eDNA has the additional benefit over trawling by being able to sample when whales are present, providing a more precise representation of their feeding ecology. By employing a multi‐marker eDNA approach, we detected 96 unique fish species across 61 families, substantially exceeding the diversity captured by concurrent trawl surveys. The higher detection rates for several potential prey taxa compared to the trawl survey suggest that the prey available to the Rice's whale may be more prevalent and more diverse than previously documented.

Recent evidence from stable isotope mixing models and prey selection indices suggest that the Rice's whale utilize high‐energy content prey, although relatively abundant prey may also contribute significantly to their diets. Previously, three fish species (*M. weitzmani, D. dumerilii*, and *A. bondi* ) and one squid genus (*Doryteuthis* sp.) were described as the most likely Rice's whale prey items based on stable isotope values. *A. bondi* in particular was identified as a key prey species due to high‐energy content and relatively high biomass (Kiszka et al. 2023). In our eDNA samples, these three fish species were also among the most prevalent and abundant taxa, occurring in 24%–95% of samples and making up 9%–52% of read abundance on average. Of the 17 fish species observed in this study with isotope signatures matching potential Rice's whale prey, eDNA yielded the same or more detections than trawling for all but 2 species, and in some cases resulted in almost four times more detections (Figure 5). Therefore, top potential prey taxa, such as *A. bondi* and *D. dumerilii*, may be more prevalent in the Rice's whale foraging habitat than the previous trawl surveys suggest (Martinez et al. 2025).

Our results also suggest that additional taxa may feature in the diet of Rice's whale. Based on eDNA‐derived relative read abundance and prevalence in the foraging habitat, we identified three additional species with matching stable isotope values as potentially contributing to Rice's whale diets ( *P. spinosus* , *B. aureorubens*, and *N. americana* ). Although occurring in only 19%–33% of samples and with lower average relative read abundances of 5%–10% (Table 2, Figure 4), both *P. spinosus* and *N. americana* have high energy densities comparable to *A. bondi* (Kiszka et al. 2023). *P. spinosus* shares a similar ecology to prey of close relatives of the Rice's whale (e.g., Bryde's whale), as it is a small (20–30 cm), schooling mesopelagic fish that often forms dense aggregations near the bottom (Best 2001; Garrison et al. 2024; Salvadeo et al. 2011). While observations from within the foraging habitat are highly valuable for characterizing potential prey availability for Rice's whale, future studies should also incorporate data and sampling from outside foraging areas in order to better determine ecological drivers of whale aggregations.

Prevalence and relative abundance data based on eDNA should be interpreted with consideration of the complex dynamics of eDNA transport in marine systems and other methodological limitations. Ocean currents can move eDNA considerable distances from its source, while temperature, UV exposure, and microbial activity affect degradation rates, potentially disconnecting detection from actual organism presence (Andruszkiewicz Allan et al. 2021; Barnes and Turner 2016; Lacoursière‐Roussel et al. 2016). Of the 41 fish species recorded from the trawl survey at these stations, 16 were not detected by either eDNA marker (Figure 2). Six of these are due to references missing from both the MiFish and Riaz reference sequence databases (Table 1). Of the remaining species that were in at least one database, two are elasmobranchs, which previous work has shown that MiFish and Riaz are not optimal at detecting (Miya et al. 2015; Stoeckle et al. 2020). Some mismatches may be due to morphological misclassification on the trawl survey, as their sister species were detected with eDNA (e.g., *Coelorinchus occa* for *C. caribbaeus* , *Ariomma melana* for *A. bondi* ). Some undetected species may be due to low sequence diversity at these markers, such as ASVs being assigned only to genus for *Pontinus*. The remaining undetected species were predominantly filefishes in the Monacanthidae family, suggesting there may be characteristics about some members of this family (e.g., low eDNA shedding rates, PCR amplification biases) that make detection with eDNA challenging for the markers used here (Gold, Shelton, et al. 2023; Thalinger et al. 2021). Despite these caveats, the differences between trawl and eDNA detections reflect both the gear selectivity of trawl sampling and the ability of eDNA to detect species even when they occur at low densities or successfully avoid capture.

While eDNA provides robust presence‐absence data and reveals previously undetected biodiversity, it has limitations in quantifying abundance. The relationship between read abundance and abundance of the organism in the area remains complex, influenced by factors including body size, DNA shedding rates, PCR amplification biases, and uneven sequencing depth (Derycke et al. 2021; Gloor et al. 2017; Sassoubre et al. 2016; Shelton et al. 2023; Silverman et al. 2021). Additionally, the present study is limited by the use of fish‐specific markers (MiFish and Riaz), while evidence from Kiszka et al. (2023) suggests that the Rice's whale may also consume mollusks, particularly squids of the genus *Doryteuthis*. Future eDNA studies in Rice's whale habitat should incorporate additional molecular markers designed to detect cephalopods and elasmobranchs, and combine eDNA metabarcoding with targeted quantitative PCR assays for key prey species to better estimate their relative abundance and distribution patterns (Shelton et al. 2022).

Our results underscore the critical need for continued development of reference sequence databases for marine taxa (Marques et al. 2021; Zhou et al. 2025). Despite our expanded reference database, which included newly sequenced voucher specimens from the trawl survey, six species captured in trawls could not be detected by eDNA due to missing reference sequences. This taxonomic gap is likely even more significant for non‐fish taxa, particularly mollusks (Weigand et al. 2019; Shi et al. 2024), which stable isotope evidence suggests may also contribute to Rice's whale diet. Coordinated efforts to expand reference databases for Gulf of America marine fauna, especially potential Rice's whale prey taxa, would substantially enhance the utility of future eDNA monitoring (Gold et al. 2021).

## 5 Conclusion

This study represents the first application of eDNA metabarcoding to characterize potential prey communities available to the critically endangered Rice's whale, demonstrating that eDNA provides a more comprehensive assessment of biodiversity than conventional methods alone. The enhanced detection of taxa previously identified as potential prey through stable isotope analysis—particularly *Parascombrops spinosus* and *Neoepinnula americana* —suggests these species may play a more significant role in Rice's whale foraging ecology than previously recognized. Implementing standardized eDNA sampling protocols alongside routine marine mammal and fisheries surveys could provide a cost‐effective means of monitoring temporal and spatial variations in prey communities, informing targeted conservation measures for this critically endangered cetacean. As anthropogenic threats continue to impact this at‐risk population, efforts to designate critical habitat for this species based on prey availability should encompass all available data, including eDNA‐derived insights (Garrison et al. 2024; Pendleton et al. 2020). Overall, improved understanding of their prey requirements and foraging ecology is essential for developing effective conservation strategies that protect both the whales and their food resources in the Gulf of America.

## Author Contributions

**Katherine Silliman:** conceptualization (equal), data curation (equal), formal analysis (lead), methodology (equal), project administration (equal), software (equal), visualization (equal), writing – original draft (lead), writing – review and editing (equal). **Lynsey A. Wilcox Talbot:** conceptualization (equal), data curation (equal), investigation (equal), methodology (equal), resources (equal), supervision (equal), visualization (equal), writing – original draft (equal), writing – review and editing (equal). **Mary Applegate:** investigation (equal), writing – review and editing (equal). **Laura Aichinger Dias:** data curation (equal), investigation (equal), writing – review and editing (equal). **Lance P. Garrison:** conceptualization (equal), data curation (equal), funding acquisition (equal), project administration (equal), supervision (equal), visualization (equal), writing – review and editing (equal). **Mark Grace:** investigation (equal), writing – review and editing (equal). **Corinne N. Paterson:** investigation (equal), writing – review and editing (equal). **Luke R. Thompson:** funding acquisition (equal), project administration (equal), resources (equal), supervision (equal), writing – review and editing (equal). **Nicole L. Vollmer:** investigation (equal), methodology (equal), resources (equal), supervision (equal), writing – original draft (equal), writing – review and editing (equal). **Patricia E. Rosel:** conceptualization (equal), funding acquisition (equal), project administration (equal), resources (equal), supervision (equal), writing – review and editing (equal).

## Conflicts of Interest

The authors declare no conflicts of interest.

## Supporting information


**Data S1:** ece372789‐sup‐0001‐Supinfo1.docx.

**Data S2:** ece372789‐sup‐0002‐Supinfo2.xlsx.

## Data Availability

Code and associated files needed to reproduce results and figures for this study are available on GitHub (https://github.com/aomlomics/rices‐prey‐edna). All vouchered 12S reference sequences have been published at the National Center for Biotechnology Information (NCBI)'s GenBank with accession numbers PV861998‐PV862034. Reference sequence databases are available on Zenodo (MiFish: https://doi.org/10.5281/zenodo.17664846, Riaz: https://doi.org/10.5281/zenodo.17727349). All 12S metabarcoding sequence data generated from this study are published at the National Center for Biotechnology Information (NCBI)' Sequence Read Archive and BioSample database with BioProject accession number PRJNA1322479. Species read count data generated from this study is published on the Ocean Biodiversity Information System (OBIS) and the Global Biodiversity Information Facility (GBIF) at https://ipt‐obis.gbif.us/resource?r=rices‐diet‐edna, following the FAIR eDNA data standards (Takahashi et al. 2025). Environmental measurements from CTD profiles, trawl catch data, and marine mammal visual observation data are also available at NCEI at https://doi.org/10.25921/52w6‐4c18.
