# Supplementary material for: Evaluating Prey Availability for the Rice's Whale (Balaenoptera ricei) Based on Environmental DNA
Source: Ecol Evol. 2026 Jan 13;16(1):e72789. doi: 10.1002/ece3.72789 (PMC12796833; doi:10.1002/ece3.72789)
Supplement: Supplementary file 1 — Data S1: ece372789‐sup‐0001‐Supinfo1.docx. [file ECE3-16-e72789-s002.docx]

# Evaluating prey availability for the Rice’s whale (*Balaenoptera ricei*) based on environmental DNA

Katherine Silliman^1,2^, Lynsey A. Wilcox Talbot^3^, Mary Applegate^4^, Laura Aichinger Dias^4,5^, Lance P. Garrison^4^, Mark Grace^6^, Corinne N. Paterson^4,3^, Luke R. Thompson^1,2^, Nicole L. Vollmer^4,3^, Patricia E. Rosel^3^

^1^Ocean Chemistry and Ecosystems Division, Atlantic Oceanographic and Meteorological Laboratory, National Oceanic and Atmospheric Administration, Miami, Florida, USA

^2^Northern Gulf Institute, Mississippi State University, Starkville, Mississippi, USA

^3^Marine Mammal and Turtle Division, Southeast Fisheries Science Center, National Marine Fisheries Service, National Oceanic and Atmospheric Administration, Lafayette, Louisiana, USA

^4^Cooperative Institute for Marine and Atmospheric Studies, Rosenstiel School for Marine, Atmospheric, and Earth Science, University of Miami, Miami, Florida, USA

^5^Marine Mammal and Turtle Division, Southeast Fisheries Science Center, National Marine Fisheries Service, National Oceanic and Atmospheric Administration, Miami, Florida, USA

^6^Tulane University Department of Ecology and Evolutionary Biology, New Orleans, Louisiana, USA



**Supp. Fig. 1.** Overlap of fish detections between trawl and eDNA surveys, separated by species, genera, and families. All stations where eDNA were collected, including those without associated trawl data, are reported. When looking across all 21 eDNA samples, including those where Rice’s whales were observed feeding, the combined use of the MiFish and Riaz primer sets identified 99 unique fish species, 109 unique genera, and 62 unique families. A total of 35 species and 40 families were detected by both primer sets, while 64 species and 22 families were detected by only one of the primer sets.


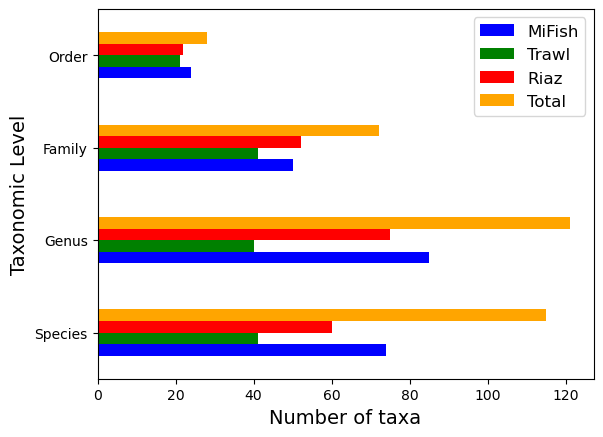


**Supp. Fig. 2.** Comparison of taxonomic richness of fish species detections between trawl and eDNA surveys. All stations where eDNA were collected, including those without associated trawl data, are reported.
